# Fe^2+^ Ions Alleviate the Symptom of Citrus Greening Disease

**DOI:** 10.3390/ijms21114033

**Published:** 2020-06-04

**Authors:** Haruhiko Inoue, Sakiko Yamashita-Muraki, Kanako Fujiwara, Kayoko Honda, Hiroki Ono, Takamasa Nonaka, Yuichi Kato, Tomoya Matsuyama, Shoji Sugano, Motofumi Suzuki, Yoshikuni Masaoka

**Affiliations:** 1 Plant Function Research Unit, Division of Plant and Microbial Sciences, National Agriculture and Food Research Organization (NARO), Institute of Agrobiological Sciences, Tsukuba, Ibaraki 305-8605, Japan; haruhiko@affrc.go.jp (H.I.); shojis@affrc.go.jp (S.S.); 2 Graduate School of Biosphere Science, Hiroshima University, Higashi-Hiroshima 739-8528, Japan; sakiko516@yahoo.co.jp (S.Y.-M.); brisbaneymyt@yahoo.co.jp (K.F.); khonda818@gmail.com (K.H.); ono-hiroki@zennoh.or.jp (H.O.); 3 Toyota Central R&D Labs., Inc., Yokomichi, Nagakute, Aichi 480-1192, Japan; nonaka@mosk.tytlabs.co.jp (T.N.); yuichi@mosk.tytlabs.co.jp (Y.K.); 4 Environment and Energy Innovation Department, Frontier Research and Development Division, Aichi Steel Corporation, Wanowari, Arao-machi, Tokai, Aichi 476-8666, Japan; t-matsuyama@he.aichi-steel.co.jp

**Keywords:** citrus greening, huanglongbing, iron nutrient, PCR, Ferroptosis

## Abstract

Citrus greening (CG) is among the most devastating citrus diseases worldwide. CG-infected trees exhibit interveinal chlorotic leaves due to iron (Fe) deficiency derived from CG; thus, Fe content is lower in infected leaves than in healthy leaves. In this study, we demonstrated that the foliar application of Fe^2+^ relieves the symptom of CG infection in citrus trees. We applied Fe^2+^ and citrate to the leaves of infected rough lemon plants. Following this treatment, a reduction in the number of yellow symptomatic leaves was observed, and their growth was restored. Using chlorophyll content as an index, we screened for effective Fe complexes and found that a high ratio of citrate to Fe^2+^ in the applied solution led to effects against CG in Shikuwasa trees. A high proportion of Fe^2+^ to total Fe was another key factor explaining the effectiveness of the solution in CG infection, indicating the importance of Fe^2+^ absorption into plant cells. We confirmed the proportion of Fe^2+^ to total Fe through the high correlation of reflectometry data via a triazine reaction and X-ray absorption fine structure analysis. These results demonstrate that the foliar application of a high-Fe^2+^ citrate solution can restore the growth of CG diseased trees.

## 1. Introduction

Citrus greening (CG), also known as *huanglongbing*, is a bacterial disease that is among the most serious citrus diseases worldwide [1,2]. CG severely affects the quality and marketability of fruit in agricultural fields. The pathogenic bacteria that causes CG is difficult to culture and spreads via contact with psyllid insects. Typical symptoms of CG-infected citrus trees include the variegated chlorosis of leaves, reduced plant height and blotchy mottle. At present, there is no effective method for preventing this disease, except by cutting down infected trees to halt the further spread of the pathogen.

CG was first documented in India in the 19th century and spread to many parts of the world until the mid-20th century [3]; it has been detected in China, Indonesia, the Philippines, South Africa, Brazil, and the United States [4,5]. The US government addressed the use of this pathogen in the Agricultural Bioterrorism Protection Act of 2002 [6]. In Japan, CG was first found on Iriomote Island in 1988 [7], and then spread to a portion of the southwestern islands of Kyushu region. Few studies have explored treatment options against a potential CG pandemic. One suggested approach for controlling CG is the injection of plant defense activators and antibiotics [8]; however, CG was found not to be susceptible to oxytetracycline in vitro.

Iron (Fe) is an essential nutrient with low solubility in neutral to alkaline soils; it is required for physiological processes in CG bacteria as well as those in citrus plants, such as photosynthesis, respiration, oxygen transport, and gene regulation [9]. Graham et al. (2017) reported that CG progressed rapidly in citrus grown in a high-pH soil condition in Florida, United States, where Fe precipitates easily in soil [10]. Similarly, CG-infected trees are not found in low-pH soils and are common in high-pH soils (>7.51) in Tokunoshima, Kagoshima Prefecture, Japan [11], suggesting that most of the CG-infected trees is recognized in the alkaline soil. Plant cultivars resistant to CG have higher root reductase activity for Fe than susceptible cultivars [12,13], and diseased leaves contain lower Fe content levels than healthy ones [14]. Gene expression analysis of the model pathogenic bacteria *Pseudomonas syringae* pv. tomato strain DC3000 in leaves of *Arabidopsis* revealed that the synthesis of bacterial siderophores to coordinate Fe transport occurs in the early stages of pathogen infection [15]. These results suggest that Fe is a main requirement for the survival of CG-causing bacteria in plants and that its virulence could be controlled by altering Fe nutrition in plants.

Fe absorption systems in plants are divided into two strategies, strategy I and strategy II [16]. Under Fe-deficient conditions, graminaceous plants release a chelator, mugineic acids to uptake Fe(III)-mugineic acids as a complex [16]. Tolerant non-graminaceous plants release reductants or chelators into the rhizosphere, enhancing proton excretion and increasing their ferric reduction capacity at the root surface and the transport of Fe^2+^ across the plasma membrane by Fe^2+^ transporters (strategy I) [17]. In contrast, some sensitive dicot plants are poorly adapted for Fe reduction. Citrus plants utilize strategy I, and some plants in the *Citrus* genus are susceptible to Fe chlorosis; citrus trees with many commercial rootstocks perform poorly in high-carbonate soils [18].

In this study, we investigated the efficacy of Fe application against CG symptoms. We performed foliar application of solutions containing Fe from various sources to plants infected by CG, and then performed polymerase chain reaction (PCR) analysis to detect the disease and evaluate plant growth. We considered the Fe valence (ferric or ferrous) in the supplied solution because Fe can be absorbed into the cytoplasm thorough potential Fe^2+^ transporters [19]. This is the first study to attempt CG eradication via plant nutrition.

## 2. Results

### 2.1. Fe Foliar Spray Alleviated CG Symptoms in Rough Lemon Citrus

To allow for more effective absorption of Fe, we then prepared an Fe ion solution (FC3); ions are transported across the cell membrane through Fe^2+^ transporters, such as a citrus iron-regulated transporter (IRT) [19]. Following the triazine reaction, the ratio of Fe^2+^ to total Fe was around 30%.

**Figure 1:**
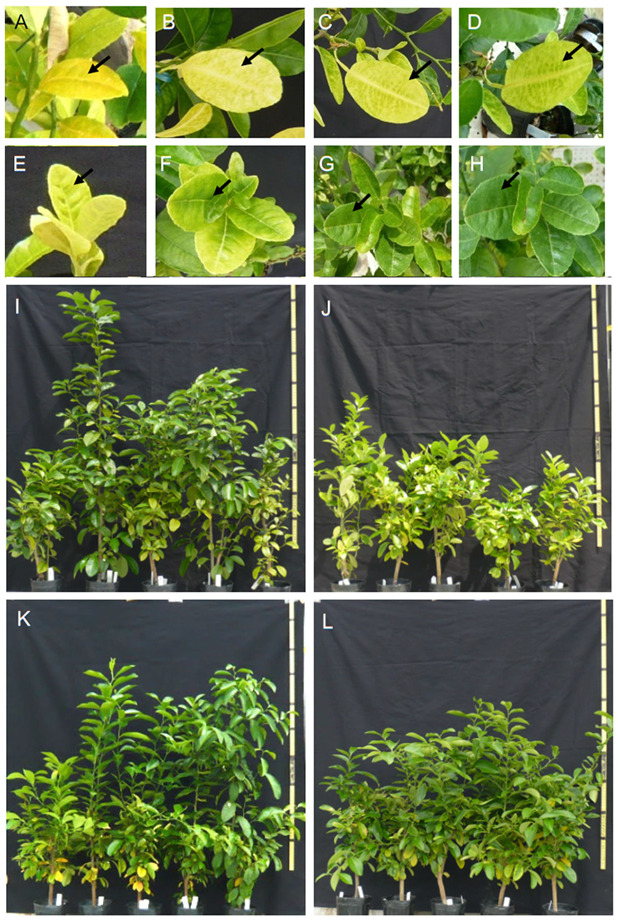
Symptom of citrus plants infected with citrus greening (CG) was relieved by foliar spraying of an Fe^2+^ solution, FC3. (**A**–**D**) Untreated CG-infected trees; (**E**–**H**) CG-infected trees treated with FC3. The arrows indicate the same leaves during the treatment period. The photographs were taken (**A**,**E**) before foliar application and at (**B**,**F**) 102 days, (**C**,**G**) 151 days, and (**D**,**H**) 192 days of treatment. Also shown are (**I**) FC3-treated CG-infected trees, (**J**) untreated CG-infected trees, (**K**) FC3-treated healthy trees, and (**L**) untreated healthy trees 192 days after the start of foliar Fe spraying.

A total of 20 citrus trees were prepared for experiments, of which 10 were infected with CG. Five infected trees received FC3 foliar spray. At 19 days after FC3 spraying, new shoots had sprouted from the basal parts of the stem, and new leaves showed rapid growth (Figure S1). In the early stages of treatment, shoots grew faster in three of the five infected plants that had received Fe foliar spray than in non-treatment groups. Infected leaves of plant not treated with Fe solution remained yellow, culminating in senescence (Figure 1A–D). In contrast, the color of infected leaves treated with FC3 spraying gradually turned from yellow to green (Figure 1E–H), even within the same leaf (arrow). Remarkable increases in plant height were observed at the early stage of treatment (Figure 1I,K), continuing for at least 4 months after treatment. The height of infected plants increased soon after the start of treatment (Figure 1I), then FC3-tereated healthy plants showed the highest growth (Figure 1K), whereas that of uninfected plants increased only during the growing season. Two infected and FC3-treated plants of the five replicates showed slower growth rates; in these trees, the Fe nutrient fertilizer effect was negligible. Infected plants that were not treated with FC3 solution showed little increase in height and produced new leaves slowly (Figure 1J).

The leaves of infected plants lost most of their green color, and blotchy mottling appeared at the start of Fe treatment. Discolored yellow leaves are a serious symptom of CG; this phenomenon is similar to signs of Fe deficiency. However, after 6 months of FC3 treatment, many parts of the leaves appeared green (Figure 1G,H). The leaf color of infected plants then returned to green, in contrast to the behavior of leaves of the uninfected control plants (Figure 1H).

**Figure 2:**
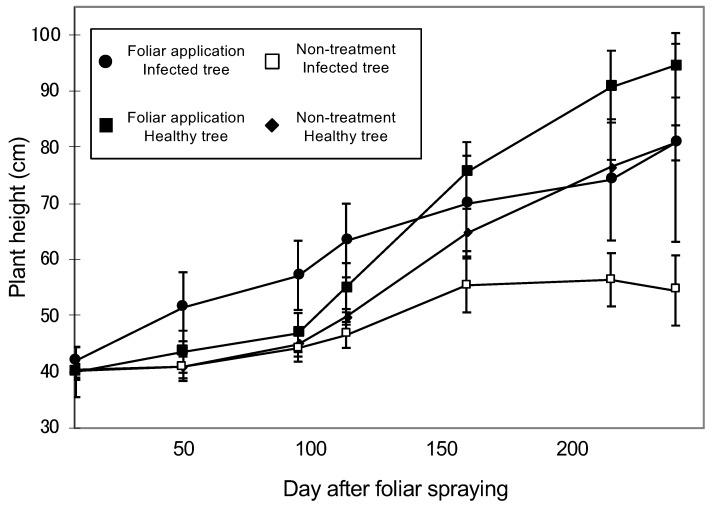
Changes in plant height among CG-infected plants treated with foliar Fe application (black circle), untreated CG-infected plants (open square), healthy plants treated with foliar Fe application (black square), and healthy untreated plants (black diamond).

Before the foliar application experiment, the plant height was around 40 cm. As the leaves return to green by applying FC3, the citrus growth of FC3-treated infected plants was higher than the other plants until 4 months (Figure 2). After that, the citrus growth with FC3-treated infected plants were similar to that of no-treated healthy plants. At 225 days after Fe application, plant height differed significantly between both healthy and infected trees receiving Fe treatment (Tukey–Kramer multiple comparison test, *p* < 0.05, Figure 2).

**Figure 3:**
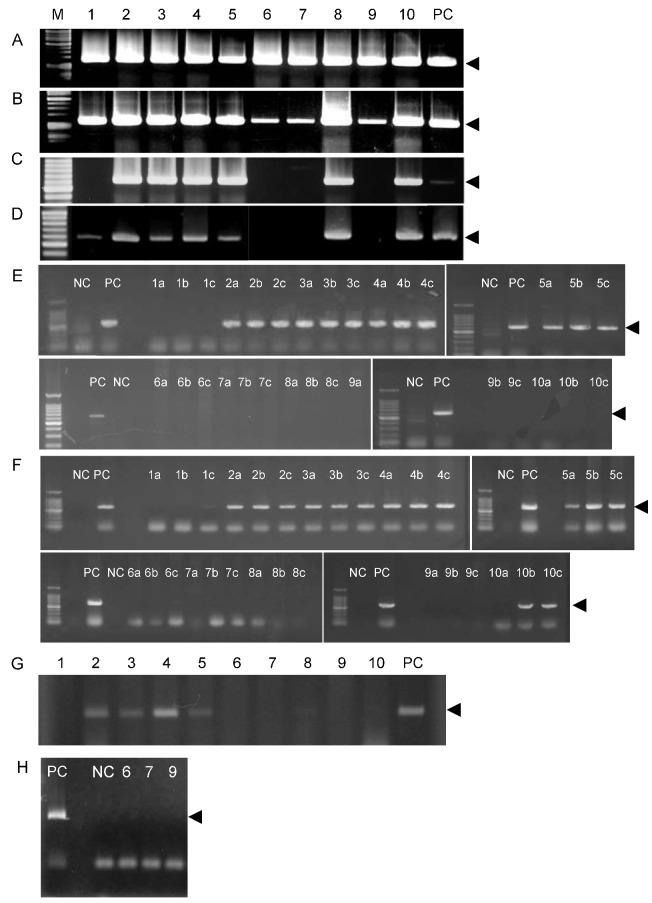
Polymerase chain reaction (PCR) amplification of the CG-bacterial disease gene. Amplified bands were separated by electrophoresis on 0.8% agarose gel. DNA from treated plant leaves was sampled (**A**) before foliar application and during treatment at (**B**) 14 days, (**C**) 54 days, (**D**) 160 days, (**E**) 192 days, (**F**) 296 days, (**G**) 1 year and 7 months, and (**H**) 1428 days. (**E**) The DNA samples were collected from leaves of infected plants in the **upper** (a), **middle** (b), and **lower** (c) portions of trees. PC: positive control. NC: healthy leaves. The arrows indicate PCR bands showing CG disease.

Before starting experimental treatments, PCR diagnosis of leaf samples showed specific bands in every infected plant (Figure 3A). The bands disappeared in the leaves of three plants treated with Fe by 54 (Figure 3C), 160 (Figure 3D), and 192 days (Figure 3E). This band also disappeared in the two remaining Fe-treated plants (#8 and #10) at 192 days after treatment (Figure 3E), although it was detected earlier (Figure 3B–D). After 1 year and 7 months, we detected no CG-related PCR bands in four of the replicate plants (#6, 7, 9, 10) and detected only a very faint band in the remaining plant (#8, Figure 3G). Finally, after 1428 days of Fe treatment, CG-derived bands were not detected in three plants (#6, 7, 9 Figure 3H), the other two plants were abruptly dead.

### 2.2. Fe Chelate Compounds Alleviated CG Symptoms

**Figure 4:**
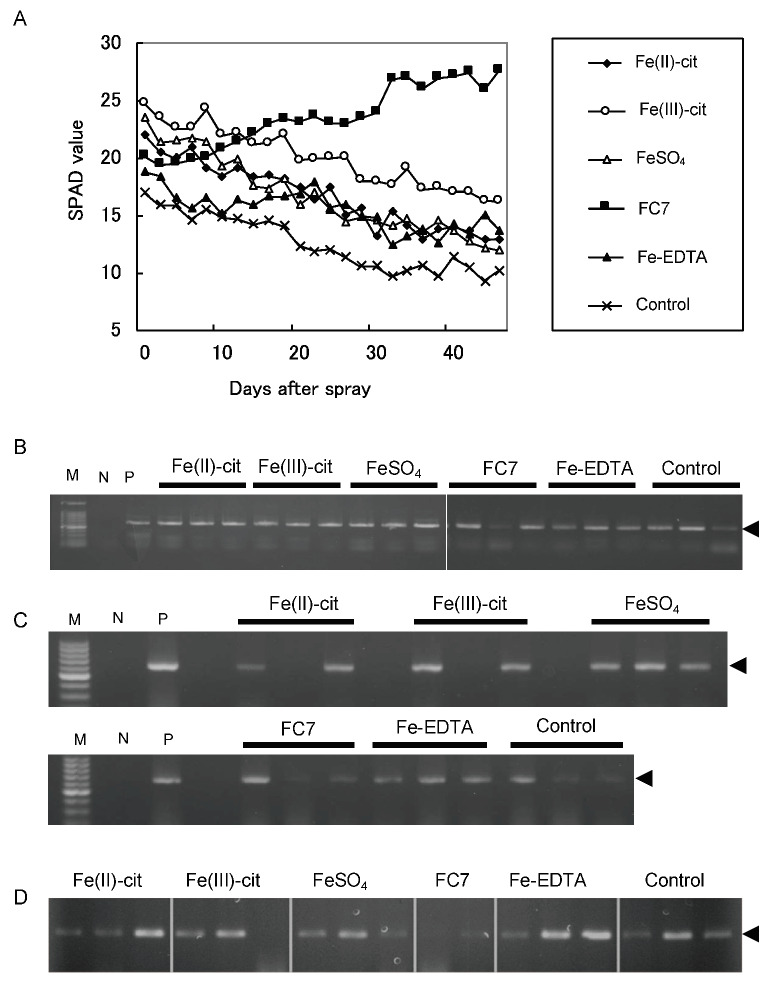
Chlorophyll index of tree leaves treated with foliar application of Fe. (**A**) Chlorophyll index of tree leaves treated with foliar application of Fe(II)-citrate (black diamond), Fe(III)-citrate (open circle), FeSO_4_ (open triangle), FC7 (black square), Fe-EDTA (black triangle), and control (cross). Infected trees (**B**) before, (**C**) 50 days after, and (**D**) 10 weeks after the foliar application of Fe(II)-citrate, Fe(III)-citrate, FeSO_4_, FC7, Fe-EDTA, and the control were sampled for genomic DNA extraction. Using these DNA samples as a template, the PCR amplification of the CG disease gene was performed. The arrows indicate PCR bands showing CG disease.

The chlorophyll value (SPAD) following FC7 treatment was 1.37-fold greater than that of recorded at the start of the experiment (Tukey–Kramer multiple comparison test, *p* < 0.05), and those for other Fe chelate compounds gradually decreased throughout treatment (Figure 4A). At 48 days after foliar application, the SPAD value was 0.58 times that for Fe(II)-citrate, 0.65 times that for Fe(III)-citrate, 0.5 times that for FeSO_4_, 0.73 times that for Fe-EDTA, and 0.6 times that of the control (Figure 4A). DNA amplification was performed to confirm CG disease in all samples at the beginning of the experiment (Figure 4B). The CG-bacterial gene was not detected in one of three samples 50 days after spraying with Fe(II)-citrate, Fe(III)-citrate, and FC7 (Figure 4C). No significant differences were detected in samples treated with FeSO_4_ or Fe-EDTA (Figure 4C). After 10 weeks of treatment, PCR amplification showed no CG-derived band in two of three samples sprayed with Fe(II)-citrate or Fe(III)-citrate, and one of three samples sprayed with FC7 or FeSO_4_ (Figure 4D). In contrast, no change was detected in samples sprayed with Fe-EDTA and water (control) (Figure 4D).

### 2.3. Ratio of Fe^2+^ to Total Fe in Solution

**Figure 5:**
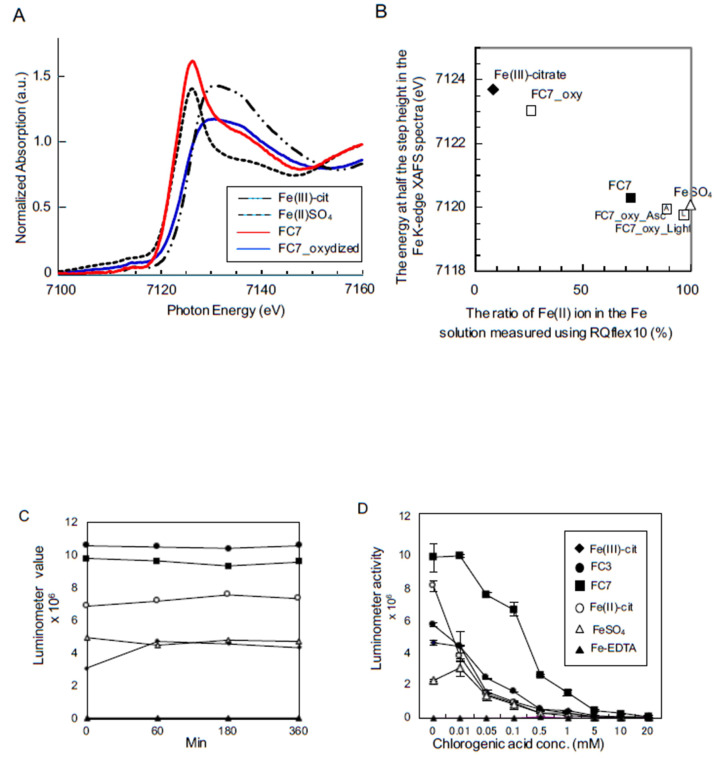
Representative Fe K-edge X-ray absorption fine structure (XAFS) spectra for Fe solutions. (**A**) The energy at half of the step height (i.e., where the normalized absorbance is 0.5) was calculated for each spectrum and (**B**) compared with the ratio measured by RQflex10 in a triazine reaction. This value is generally used as a rough estimate of the average oxidation state of Fe ions. (**C**) The time series of luminol values for six iron nutrient aqueous solutions. Fe(III)-citrate (black diamond), FC3 (black circle), FC7 (black square), Fe(II)-citrate (open circle), FeSO_4_ (open triangle), and Fe-EDTA (black triangle). (**D**) The identification of radical oxygen species in Fe^2+^ nutrient solutions using chlorogenic acid as a radical scavenger.

To confirm whether our reflectometry results accurately determined the Fe^2+^ content of the foliar application solution, we performed XAFS analysis using a highly concentrated Fe solution and compared the results with those of reflectometry analysis of the diluted Fe solution. Figure 5A shows representative Fe K-edge XAFS spectra for the Fe solutions. The energy at half of the step height (i.e., where the normalized absorbance is 0.5) was calculated for each spectrum (Figure 5B). This value is generally used as a rough estimate of the average oxidation state of Fe ions. We confirmed that the ratios of Fe^2+^ in the Fe solutions obtained by triazine reaction calculations were highly correlated with those determined by XAFS analysis, strongly indicating that the Fe^2+^ ratio of the foliar application solution could be important for alleviating CG symptoms. Interestingly, sunlight exposure increased the Fe^2+^ ratio in oxidized FC7 from 25% to 97%, also adding ascorbate as a reductant (up to 89%), suggesting that foliar application of FC7 to citrus trees in the field in sunlight is highly effective for mitigating CG disease symptoms.

### 2.4. ROS Generation in Fe Solutions

The chemiluminescence results show evidence of reactive oxygen species, ROS generation (Figure 5C) in all Fe nutrient solutions except Fe-EDTA, continuing for at least 360 min. FC3 and FC7 had remarkably high luminometer readings, indicating that these Fe nutrients generate large amounts of ROS. Luminometer readings decreased as chlorogenic acid concentration increased; therefore, the dominant product was a hydroxyl radical (Figure 5D). Hydroxy radical generation continued in FC3 and particularly in FC7 at higher chlorogenic acid concentrations.

## 3. Discussion

Fe has many functions in plants; it plays indispensable roles in various physiological processes, such as photosynthesis, respiration, oxygen transport, and gene regulation [9]. However, in many soil environmental conditions, such as calcareous soils, it is difficult for plants to absorb Fe. We developed FC3 and FC7, an Fe^2+^ nutrient additive solution containing a stable form of Fe^2+^. FC3 and FC7 thus facilitate Fe absorption by plants (Figure 1, Figure 2 and Figure 4A). The mechanism of Fe absorption in leaves remains unclear; however, the results of this study indicate that the foliar application of Fe^2+^ was more effective than that of Fe-EDTA (Figure 1), indicating that plants absorb Fe^2+^ through Fe^2+^ transporters, such as IRT, in leaves. Despite the routine foliar application of micronutrients in horticulture, little is known about the roles of metal transporters in citrus. Recently, Zn/Fe-regulated transporters (ZIPs) were identified in a genome-wide study of functional complementation in *Poncirus trifoliata* using yeast [20]. In leaves, *PtZIP7* and *PtIRT1* expression is highly upregulated in response to Fe deficiency [20] and *PtIRT3* is constitutively expressed in all tissues [21], indicating that transporters such as the ZIP family are responsible for Fe^2+^ absorption through plant shoot parts. The results of our experiments showed that the plant growth with foliar application of FC7 to infected plants were accelerated (Figure 3), indicating that Fe^2+^ is the preferable form for absorption in leaves, via Fe^2+^ transporters. Furthermore, sunlight exposure increased the ratio of Fe^2+^ in FC7 solution, indicating that the foliar application of FC7 in the field is the optimal pathway for Fe acquisition by citrus trees (Figure 5).

A few studies have explored the roles of micronutrients in CG-infected plants, despite leaf color changes indicative of micronutrient deficiency. Typical symptoms of infected trees include yellow chlorotic leaves with a mottled, blotchy appearance and variegated chlorosis [5]. Decreased Fe content has been previously detected in the leaves of CG-infected plants [22]. Recently, CG infection was found to be highly correlated with the soil pH within the same island [11], which is consistent with the findings that soil pH management reduces CG infection [10] and that CG-tolerant plant cultivars have higher root reductase activity for Fe than susceptible cultivars [12]. Together, these findings strongly suggest that CG infection is related to Fe nutrition in citrus trees. CG-infected plants also cause Zn deficiency [14,23], and zinc treatment has been reported to alter microbiome-producing siderophores in the midribs of CG-affected citrus trees. Fujiwara et al. (2018) reported that treatment with antibiotics, such as oxytetracycline, did not directly affect CG pathogens in vitro, but that oxytetracycline treatment changed the CG-related microbiota, resulting in decreased CG symptoms and suggesting that phloem-inhabiting CG pathogens exchange benefits with CG-associated microbes to colonize the host and display pathogenicity [24]. Siderophores from neighboring organisms have been shown to depend on the growth of other bacteria [25]. It has long been recognized that siderophores act as key virulence factors in the host [26,27]. However, plant Fe and Zn are coordinated by nicotianamine in downstream phytosiderophores, such as 2′-deoxymugineic acid (DMA), in the phloem [28]. Nicotianamine is a ubiquitous plant-derived chelator whose biosynthesis genes are expressed in the phloem [29,30,31]. Recently, DMA was also found in olive trees [32]. Their complexes are transported through specific yellow stripe-like (YSL) family transporters that function in the phloem to translocate metals to required tissues [33,34,35,36]. Thus, the homeostasis of micronutrients, including the phloem microbiota, involve competition for Fe between pathogenic bacterial siderophores and phytosiderophores.

The restored growth of CG symptoms in citrus plants following Fe application in this study was likely caused by improved Fe nutrition, which allowed for the induction of the proper defense response to the disease. The foliar application of divalent Fe was therefore very effective in CG-infected trees in this study. Recent research on plant immune responses to Fe and pathogenic bacteria has shown significant progress, leading to the identification of responsible genes at the molecular level. In *Arabidopsis*, bacterial siderophores produced from *Erwinia chrysanthemi* (reassigned as *Dickeya dadantii*) were shown to enhance the expression of the Fe storage gene *Ferritin1* (*FER1*); mutant *fer1* plants are susceptible to *E. chrysanthemi* [37]. The susceptibility of *Arabidopsis* to *D. dadantii* increased in a metal transporter mutant, *irt1* [38]. Susceptibility to the pathogen *Pseudomonas syringe* pv. tomato DC3000 was enhanced in the YSL Fe transporter mutant *ysl3* compared with wild-type plants [39]. The induction of rice defense genes has been shown to increase, offering enhanced resistance to *P. oryzae* infection, in rice plants grown at high Fe levels [40]. A comprehensive expression analysis showed that the expression of Fe reductase oxidase genes *FRO2*, *FRO7*, and *FRO8* was partially suppressed in CG-infected citrus [41].

Future studies should investigate the relationship between nutrition and CG. Complete pathogen control should be attempted to prevent infection and cure infected trees through citrus plant nutritional physiology and plant pathology.

## 4. Materials and Methods

### 4.1. Preparation of Infected Plant Materials and Fe Nutrition

We selected 2-year-old infected and healthy rough lemon (*Citrus jambhiri* Lush.) plants as sources for bud sticks. Infected plants harbored a strain collected from Ishigaki Island, Ishi-1, which we used as an inoculum source. We collected bud sticks from source plants and side-grafted them to stems of recipient plants at a height of about 50 mm, after which the trees were grown for 2 years. A total of ten infected and ten healthy, uninfected plants were prepared, with five replications per plant. The plant height was around 40 cm at the start of Fe^2+^ treatment and increased during the later stages of the experiment.

All plants were obtained with the collaboration of the National Institute of Fruit Tree Science and the Okinawa Prefectural Agricultural Research Center. Plants were transferred to 1-L plastic pots (one plant per pot) filled with a commercial mix of soil and peat moss (Ikubyou Baido, Takii & Co. Ltd., Kyoto, Japan). Plant pots were placed in a greenhouse for 6 months at 32 °C during the day and 28 °C at night. Each pot was supplied with 50 mL of nutrient solution containing 10 mM Ca(NO_3_)_2_, 1.5 mM KH_2_PO_4_, 2.5 mM MgSO_4·_7H_2_O, and 1 mM K_2_SO_4_ at 10-day intervals during treatment. We collected three to five leaves (usually the fourth or fifth leaf from the top of a branch) from each plant for the detection of the CG-bacterial gene by PCR. We applied Fe solution containing a 1:3 ratio of Fe(II)-citrate (FC3) (Aichi Steel Corporation, Aichi, Japan) to five infected plants and five uninfected plants, and used the remaining plants as controls. After confirming infection by PCR, we supplied commercial FC3 nutrient fertilizer to the plants at 5-day intervals, and then diluted the treatment solution concentration to 15 mg/L Fe. Surfactant (Kao Corporation, Tokyo, Japan) was added to the solution (0.01%, *v*/*v*), and then 50 mL was sprayed on the entire leaf surface. Control plants were supplied with water containing the same concentration of surfactant. Treatment was continued for 1 year and 7 months.

We performed two sets of trails for the growth test. CG-infected Shikuwasa plants (*C. depressa*) were kindly provided by the National Institute of Fruit Tree Science and the Okinawa Prefectural Agricultural Research Center. Each pot was supplied with 50 mL of nutrient solution, as described above at 5-day intervals during treatment. After confirming infection by PCR, Fe solution containing a 1:7 ratio of Fe(II)-citrate (FC7) was diluted to 15 mg/L Fe, and 50 mL was sprayed on the entire leaf surface every other day. To produce a more effective Fe complex for foliar application against CG, we mixed FC7, which contained a ratio of about 70% Fe^2+^ to 30% total Fe, and examined the effects of this Fe solution when dissolved in Fe(II)-citrate, Fe(III)-citrate, Fe(II)SO_4_, or Fe^3+^-EDTA.

### 4.2. Colorimetric Analysis of Fe Solutions

To gain chemical evidence of how stable in FC3 and FC7 solutions Fe^2+^ is, the concentrations of Fe^2+^ and total Fe were measured following the colorimetric method. Briefly, Fe-containing solutions were diluted with water to a target concentration of 0.5–20 mg/L, and the Fe^2+^ in the solution was reacted with triazine derivative to form a red–violet-colored complex. The solutions were analyzed using an RQflex10 reflectometer (Merck KGaA, Darmstadt, Germany) to quantitate the Fe^2+^ concentrations in each. The solutions were then subjected to the Reflectoquant Iron Test (Merck KGaA, Darmstadt, Germany). Ascorbate acid was added to the solution as a reductant, and the solution was allowed to set for 1 min; the solutions were then analyzed using the RQflex10 reflectometer to determine the total Fe concentrations.

### 4.3. Oxygen Radical Determination

We then performed three chemiluminescence experiments. Six different Fe nutrients, a 1:1 ratio of Fe(II)-citrate, Fe(III)-citrate, FC3, FC7, FeSO_4_, and Fe-EDTA, were individually diluted to 15 mg/L Fe using distilled deionized water, and 100 mL of the aqueous solution of each Fe nutrient was incubated for 60, 180, or 360 min at room temperature. We reacted 50 μL of the aqueous solution of each Fe nutrient with 50 μL of luminol solution; the reaction was automatically measured by chemiluminescence for 10 s (Luminosenser-PSN.AB-2200; ATTO Co., Ltd., Tokyo, Japan). To identify reactive oxygen species (ROS), chlorogenic acid was used to detect the presence of hydroxyl oxygen radicals [42]. Aqueous solutions containing 1.5 µg/100 µL of each Fe nutrient (Fe(II)-citrate, FC3, FC7, Fe(III)-citrate, and FeSO_4_) were reacted with 50 µL of 0.01, 0.05, 1, 5, 10, or 20 mM chlorogenic acid solutions and the measured chemiluminescence. The stability of the determined Fe^2+^ concentrations was confirmed by 24 h of observation with a luminometer, following the luminol method [43].

### 4.4. X-ray Absorption Fine Structure (XAFS) Analysis

The Fe solution was diluted to approximately 500 mg Fe/L, and placed in a plastic bag for XAFS analysis.

XAFS measurements were performed at the Super Photon ring-8 BL33XU beamline in Hyogo, Japan [44,45]. A silicon channel-cut crystal cooled with liquid nitrogen was used to monochromatize the X-rays. Higher harmonics of the X-rays were rejected using a pair of rhodium-coated silicon mirrors. The beam size at the sample position was 1 mm (height) × 2 mm (width). Fe K-edge XAFS spectra were collected in transmission mode over a period of 17 min.

### 4.5. CG-Bacterial DNA Extraction and Detection by PCR

Leaves were washed with distilled water and blotted to remove moisture. Each leaf was cut in half lengthwise, the midrib part removed, and the remaining part of the leaf macerated and flash frozen using liquid nitrogen. Frozen leaves were homogenized using a mortar and pestle without buffer and incubated at 65 °C with 5 mL of CTAB buffer solution (1% *v*/*v* CTAB, 50 mM Tris-HCl, pH 8.0, 0.7 M NaCl, 10 mM EDTA) for about 30 min; 5 mL of chloroform/isoamyl alcohol (24:1, *v*/*v*) was then added and the sample was mixed for a further 30 min at the same temperature. The solution was centrifuged at 3000 rpm for 15 min and the supernatant was transferred to a 50 mL tube. This treatment was repeated three times to remove protein. A one-tenth volume of 10% (*v*/*v*) CTAB with 0.7 M NaCl was added to the supernatant and mixed, and the same volume of CTAB solution without NaCl was added, mixed gently overnight, and centrifuged (1800 rpm, 10 min); the supernatant was then removed. The pellet was suspended in 1 mL of precipitant buffer solution (1 M NaCl, 50 mM Tris-HCl, 10 mM EDTA) and nucleic acid was precipitated by adding the same volume of isoamyl alcohol, centrifuged at 1800 rpm for 10 min, and the supernatant was removed. A 70% (*v*/*v*) volume of ethyl alcohol was added to wash the pellet and the inside surface of the tube. A 2-mL aliquot of 70% (*v*/*v*) ethyl alcohol was added to wash the inside surface and the precipitant was centrifuged at 3000 rpm for 5 min. The supernatant was removed, and DNA was dissolved in 200 µL of TE (1 mM Tris-HCl, 0.1 mM EDTA) and stored until use.

We performed PCR to amplify a portion of the CG-bacterial gene cluster using primers with the sequences 5′-GTGTCTCTGATGGTCCGTTTGCTTCTTTTA-3′ (MHO0353) and 5′-GAACCTTCCACCATACGCATAGCCCCTTCA-3′ (MHO0354) as described previously [46]. The PCR reaction mixture (20 µL) consisted of 1 µL MgCl_2_ (2.4 mM final concentration), 10 µL TaKaRa Premix Taq 2 × PCR solution (TaKaRa Bio, Inc., Shiga, Japan), 1 µL (90 ng) of each primer solution, and an arbitrary amount of DNA template (approximately 20 ng). Amplification was performed using an MJ Mini Thermal Cycler (#PTC-1148; Bio-Rad Laboratories, Inc., Hercules, CA, USA); the PCR program was initialized at 94 °C for 3 min, followed by 35 cycles of 94 °C for 60 s, 68.5 °C for 60 s, and 72 °C for 180 s, with a final step of 72 °C for 3 min before holding at 4 °C until use. The amplified 630 bp fragment derived from CG-causing bacteria was separated by 0.8% agarose gel electrophoresis.

## Supplementary Materials

The following are available online at https://www.mdpi.com/1422-0067/21/11/4033/s1. Figure S1: Numbers of new leaves from citrus plants affected by citrus greening (CG) 19 days after the foliar spraying of FC3 solution.
