# Supplementary material for: Fe2+ Ions Alleviate the Symptom of Citrus Greening Disease
Source: Int J Mol Sci. 2020 Jun 4;21(11):4033. doi: 10.3390/ijms21114033 (PMC7312295; doi:10.3390/ijms21114033)
Supplement: Supplementary file 1 [file ijms-21-04033-s001.pdf]

Supplementary Marterials

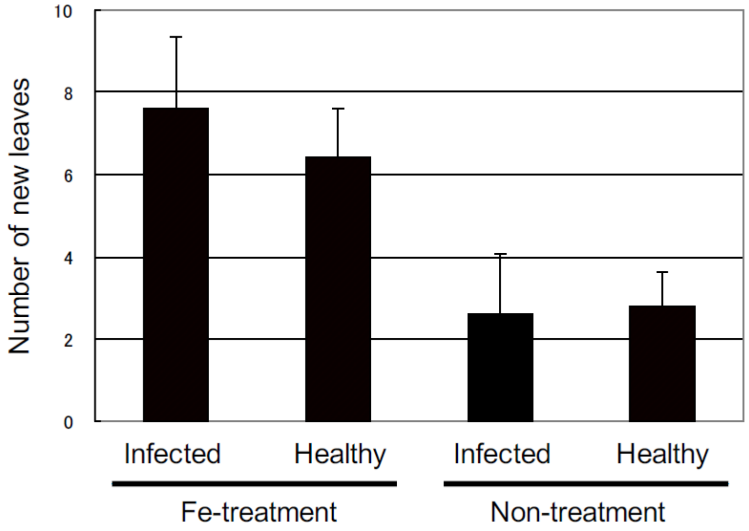

Figure S1. Numbers of new leaves from citrus plants affected by citrus greening (CG) 19 days after the foliar spraying of FC3 solution.
